# Social determinants of involuntary psychiatric hospital admissions in Ontario, Canada

**DOI:** 10.1192/j.eurpsy.2024.692

**Published:** 2024-08-27

**Authors:** K. P. Fung, S. Kim

**Affiliations:** ^1^University of Toronto, Toronto; ^2^McMaster University, Hamilton, Canada

## Abstract

**Introduction:**

In Ontario, Canada, patients may be admitted to the hospital involuntarily if they are deemed to be suffering from symptoms of a mental disorder that may result in imminent serious bodily harm to themselves or others, or that may cause serious physical impairment to themselves (e.g., inability to keep themselves safe and warm in the winter). This measure can be life-saving. However, in addition to ethical and human rights considerations, resorting to coercive admissions may be an indication that those who are suffering from mental illness are not able to access or receive timely and appropriate intervention. While recent studies have suggested that the rate of involuntary hospital admission may be increasing, data on social determinants of involuntary hospital admissions are limited.

**Objectives:**

We examined social factors associated with involuntary admissions using a Canadian provincial database.

**Methods:**

Binary logistic regression models were conducted to examine the associations between social factors (low income, indigeneity, rurality, housing type) and involuntary admissions, controlling for age, sex, and psychiatric diagnoses. Data from March 2019 to March 2021 was extracted from the Ontario Mental Health Reporting System admission dataset, comprising of a sample of 9,848 patients admitted to eight psychiatric hospitals in Ontario. Odds ratios and 95% confidence intervals are reported.

**Results:**

In 2021, the proportion of involuntary patients increased significantly by 6.8 percentage points to 55.7% compared to the previous year (48.9%). Indigenous status (First Nations, Metis, Inuit) [1.75 (1.38-2.21) **], living in rural areas [2.78 (2.48-3.12)], living in assisted residence [1.41 (1.21-1.64) **], homelessness [1.63 (1.38-1.91) **], male sex [1.21 (1.10-1.33) **] and younger age [0.99 (0.98-0.99) **] were associated with involuntary admissions, while income was not a significant factor. Compared to a diagnosis of a psychotic disorder, substance use disorders [0.11 (0.10-0.13) **] and mood and anxiety disorders [0.32 (0.29-0.36) **] showed decreased odds of involuntary admission, while neurocognitive disorders increased the odds of involuntary admission [3.86 (2.91-5.11) **].

**Conclusions:**

Consistent with other findings, involuntary psychiatric hospital admissions in ON, Canada, have increased recently, which may in part be related to the pandemic. Rurality, indigenous status, and unstable housing have been found to be associated with involuntary admissions. The study findings support the need for better preventive and intervention strategies to serve vulnerable psychiatric patients, including addressing the social determinants of health such as housing, and increasing access to culturally competent and safe community-based mental health supports and services.

**Disclosure of Interest:**

None Declared

